# Combination Immunotherapy With Radiotherapy in Non‐Small Cell Lung Cancer: A Review of Evidence

**DOI:** 10.1002/cam4.70402

**Published:** 2024-11-11

**Authors:** Justin L. Burr, Kurtis C. Johnson, Joseph J. Carmicheal, Chi Lin, Apar Kishor Ganti

**Affiliations:** ^1^ Department of Radiation Oncology University of Nebraska Medical Center Omaha Nebraska USA; ^2^ Division of Hematology‐Oncology, Department of Internal Medicine University of Nebraska Medical Center Omaha Nebraska USA

## Abstract

**Background:**

Radiotherapy plays a fundamental role in the treatment of patients with all stages of non‐small‐cell lung cancer (NSCLC). The emergence of immune checkpoint inhibitors (ICIs) has transformed the standard of care in these patients. The use of ICIs is increasingly utilized in the definitive setting as an adjunct to chemoradiotherapy or surgery and remains a vital component in the treatment of metastatic disease. Despite improvements in patient survival, the use of immunotherapy as monotherapy has shown limited overall response rates with susceptibility to resistance. Radiotherapy has been identified as a viable option to enhance the response rate to ICI and improve outcomes in NSCLC.

**Methods:**

We queried the English PubMed database utilizing variably combined search items including “radiation,” “chemoradiation,” “immune checkpoint,” “immunotherapy,” “stereotactic body radiotherapy,” and “non‐small‐cell lung”. We additionally searched various acceptable alternative terms for similar keywords such as “radiotherapy” in place of “radiation.” These results were subsequently curated for relevance and impact on current treatment paradigms.

**Results:**

In this review, we discuss preclinical and clinical studies relating to combinatorial use of immunotherapy and radiation in NSCLC. These studies are presented in the context of early‐stage, operable stage III, unresectable stage III, and metastatic disease. The majority of the data illustrate promising results regarding the additive or synergistic effects of radiation and immunotherapy with a suggestion that the timing of these treatment modalities is crucial to optimizing outcomes.

**Conclusion:**

While there is now evidence regarding the favorable interplay between radiation and immunotherapy in NSCLC, there remain multiple unanswered questions which are expected to be addressed in ongoing clinical trials.

## 1 Introduction

Cytotoxic chemotherapy and radiation have long been mainstays in the management of patients with non‐small‐cell lung cancer. However, since first report of durable responses to nivolumab in heavily pretreated patients with advanced non‐small‐cell lung cancer (NSCLC) [1], immune checkpoint inhibitors have revolutionized the management of NSCLC. The benefits of combining cytotoxic chemotherapy with immunotherapy have been demonstrated in patients with advanced NSCLC [2, 3, 4, 5].

**FIGURE 1:**
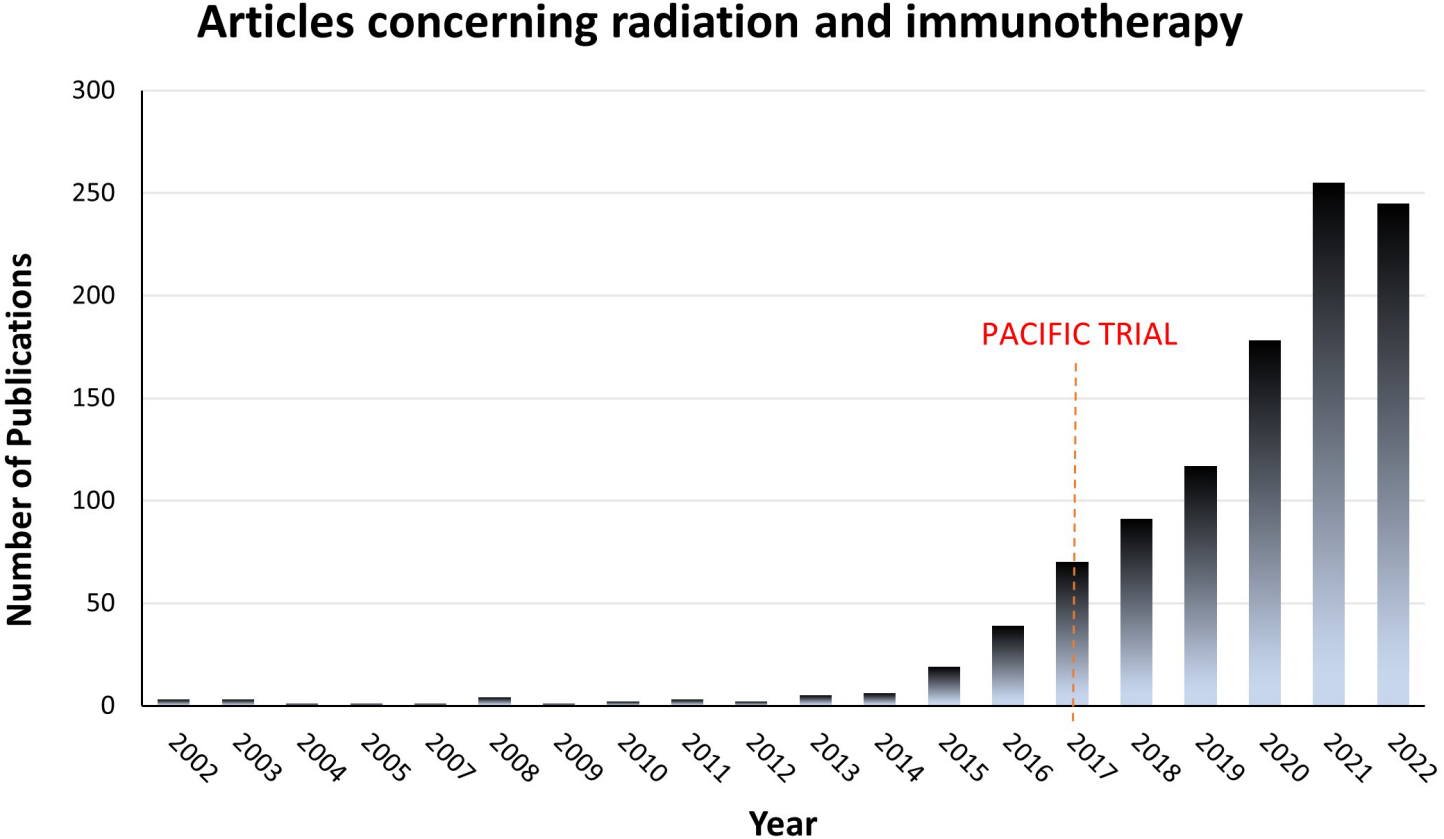
A PubMed literature search was completed with the keywords “Combination Radiation and Immunotherapy.” This bar graph depicts the number of research articles pertaining to this topic by year of publication. A dramatic increase in the number of articles has occurred in recent years which is also illustrated relative to 2017: The year data from the seminal PACIFIC trial was first published.

Recent trials have demonstrated a benefit of the addition of immunotherapy in operable and nonoperable settings [6, 7]. Radiotherapy continues to represent a vital component when treating patients with early‐stage, advanced‐stage, and metastatic NSCLC. There have been several anecdotal reports on the possible benefits of the combination of radiotherapy with immunotherapy demonstrating out‐of‐field tumor responses (abscopal effects) [8, 9, 10]. However, long‐term data from larger studies exploring this phenomenon in lung cancer patients has been considered largely lacking [11]. These discoveries, among others, have led to numerous trials investigating the role of combinatorial radiation and immunotherapy. This now represents a growing and intriguing topic resulting in a dramatic increase in the medical literature regarding the subject in recent years (Figure 1). Congruently, there have been many encouraging results from recent trials in patients with all stages of non‐small‐cell lung cancer. The following review examines the current data on the interplay between radiation and immune checkpoint inhibitor therapy in non‐small‐cell lung cancer. We queried the English PubMed database utilizing variably combined search items including “radiation,” “chemoradiation,” “immune checkpoint,” “immunotherapy,” “stereotactic body radiotherapy,” and “non‐small‐cell lung”. We additionally searched various acceptable alternative terms for similar keywords such as “radiotherapy” in place of “radiation.” These results were subsequently curated for relevance and impact on current treatment paradigms.

## 2 The Evolution of Immune Checkpoint Inhibition

After the discovery of the T‐cell receptor in 1982 [12], Allison, McIntyre and Bloch demonstrated the co‐inhibitory action (i.e., cell surface receptor regulation of T‐cell activation) of cytotoxic T‐lymphocyte‐associated protein‐4 (CTLA‐4). They showed that blockade of CTLA‐4 leads to enhanced antitumoral response in mouse models [13]. Similarly, Ishida et al. discovered that programmed cell death protein‐1 (PD‐1) was implicated in programmed cell death of T‐lymphocytes [14]. Later, it was found that PD‐1 had a role as an immune checkpoint and indeed was another co‐inhibitory receptor [15]. Subsequent discoveries revealing tumors escape host immune response with overexpression of its receptor ligand, programmed death‐ligand 1 (PD‐L1), led the way for Iwai et al. to demonstrate that the inhibition of the PD‐1/PD‐L interaction may lead to immunogenic tumor suppression [16]. These findings ushered in a new era in systemic therapy allowing practitioners to offer noncytotoxic treatment options which utilize the host's own immune system to target tumor cells.

Immunotherapies targeting T‐cell checkpoints were first evaluated in the metastatic setting and their use has become a cornerstone of systemic therapy in those with advanced disease. Initial trials in various malignancies evaluating immune checkpoint inhibitor (ICI) therapy showed promising results with durable response, however, the overall response rate (complete and partial response) to monotherapy for anti‐CTLA‐4, anti‐PD‐1, and anti‐PD‐L1 appears to be in the range of 15%–45% [6, 17, 18]. Immune escape mechanisms including decreased antigen presentation, an immunosuppressive tumor microenvironment, and upregulation of immune checkpoint pathways in NSCLC have been described and contribute to these relatively low response rates [19]. Primary and acquired resistance to ICI therapy has been demonstrated and several pathways for overcoming this resistance have been proposed, including combinatorial therapies involving ICI utilization with tumor vaccination, chemotherapy, and radiotherapy [19, 20, 21, 22, 23, 24, 25].

## 3 Radiotherapy and the Immune Milieu

Radiation‐induced tumor cell death releases tumor‐associated or tumor‐specific antigens which can then be targeted by the host immune system [26]. This increase in antigen availability can help ameliorate cancer's innate decrease in antigen presentation, thus “unmasking” the cell, facilitating immune recognition and attack. Concurrently, radiation causes release of damage‐associated molecular patterns (DAMPs) from dead and dying cells, for example, heat shock proteins, cell‐free DNA, RNA, ATP, histones, etc. [27] The release of DAMPs activate various innate immune cells including dendritic cells [28]. CD80 and CD86 found on the surface of activated dendritic cells can bind to CD28 or CTLA4 on the surface of T‐cells to activate or suppress the immune response, respectively [29]. Additionally, radiation increases expression of MHCs, adhesion molecules, and death receptors, which bolsters antitumoral immune activity [30, 31]. Radiation has also been shown to activate the Stimulation of Interferon Genes (STING) pathway which is an incredibly potent immune cell activator [26]. These radiation‐induced changes on the tumor surface and in the tumor micro‐environment have the ability to potentiate the innate and adaptive immune systems' ability to engage and destroy tumor cells [32].

## 4 Radiotherapy and Checkpoint Inhibition

**FIGURE 2:**
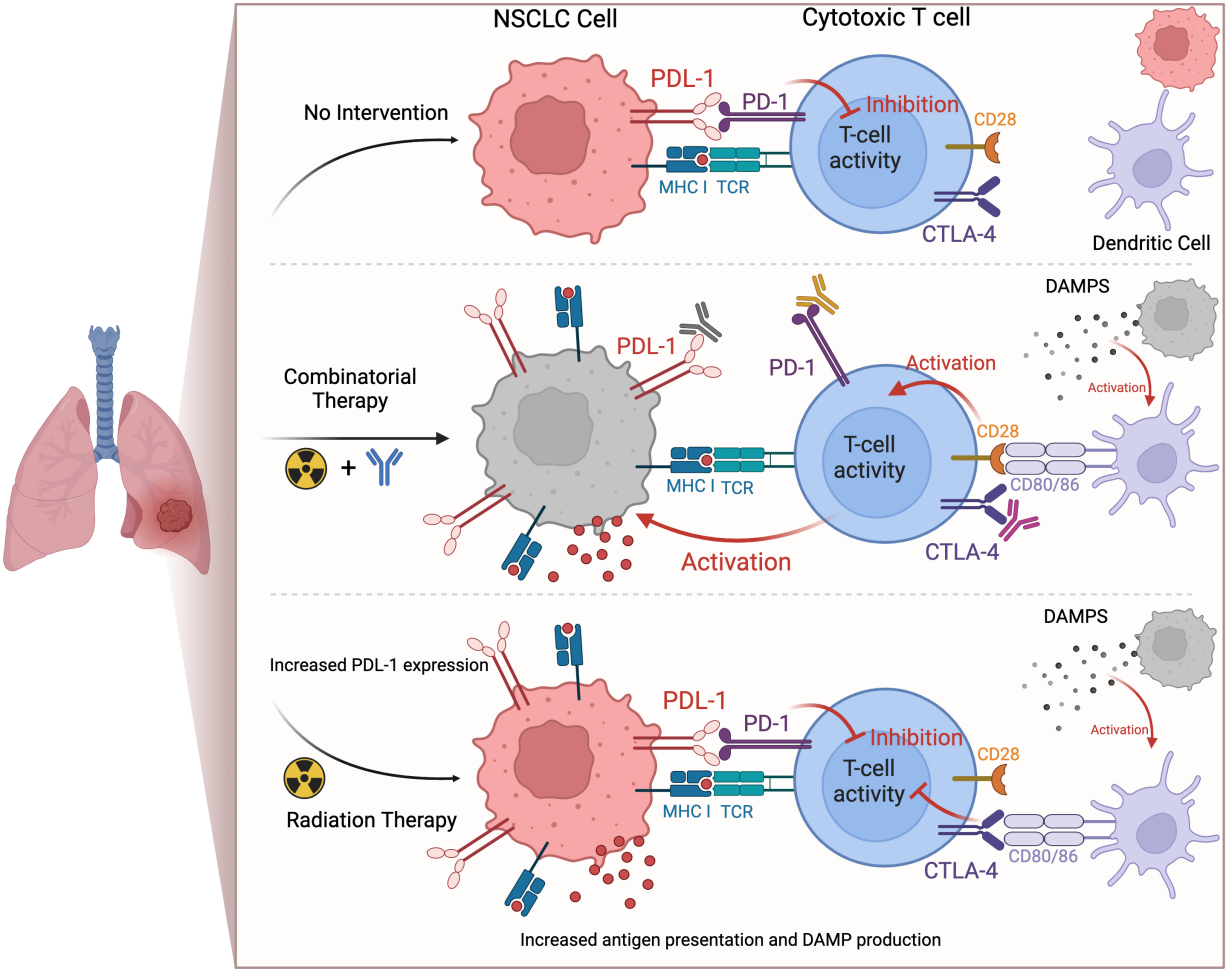
Cartoon depiction of the plausible synergistic effects of combinatorial radiation and immunotherapy. The top panel depicts tumor cell interaction with a cytotoxic T‐cell. Even with some antigen availability and presentation, T‐cell activity is suppressed via PD‐1 and CTLA‐4. A nonactivated dendritic cell is also shown as present within the tumor milieu. The bottom panel depicts the example effects radiation monotherapy has on the tumor cell which includes an increase in immunogenicity via antigen release and presentation as well as activation of dendritic cells via the release of DAMPs from dead and dying cells. Activated dendritic cells can subsequently inhibit or activate T‐cell function via CD80/86 binding with CTLA‐4 or CD28, respectively. Additionally, radiation can also cause an upregulation of PDL‐1 which in turn inhibits T‐cell activation via interaction with PD‐1. The middle panel depicts the addition of checkpoint inhibitors which may act synergistically with the increased antigen release/presentation and dendritic cell activation facilitated by radiation therapy. Blockades with antibodies directed to PDL‐1, PD‐1, and CTLA‐4 prevent the inhibition of T‐cell activation, thereby enhancing tumor‐killing potential.

While the exact mechanism is unknown, given the aforementioned immunogenic implications of radiation, it is a reasonable conclusion that radiotherapy has the potential to stimulate an immune response through T‐cell activation. Despite this activation, the presence of inhibitory molecules including PD‐1 and CTLA4 on the surface of T‐cells, overexpression of their respective ligands by tumor cells, and interaction with DAMP‐activated dendritic cells all work in tandem to suppress T‐cell function [29, 32]. Thus, the addition of immune checkpoint inhibitor therapy to radiation treatment could help prevent T‐cell inhibition and amplify tumor killing by the adaptive immune system (Figure 2). This notion is corroborated by preclinical findings that radiation facilitates the infiltration of T‐cells into the tumor tissue and subsequent T‐cell activation can be enhanced with the addition of PD‐1 blockade [33].

In direct counterpoint to the above studies, ionizing radiation is known to induce release of immunosuppressive cytokines as well as have an anti‐immunogenic effect given the relative radiosensitivity of immune cells [26]. Unfortunately, the dose–response relationship of radiation to induce adequate and sustained T‐cell activation is uncertain. There are conflicting results regarding the optimal dose for inducing meaningful immunogenic responses and a multitude of dose and fractionation regimens of radiotherapy have been proposed depending on the specific clinical scenario in which it will be combined with immunotherapy [34].

For example, one study with NSCLC mouse models showed that low‐dose radiation (2 Gy × 1) had profound impacts on the local recruitment of T‐cells and dendritic cells to the primary site of radiation, whereas high‐dose radiation (8 Gy × 3) led to increased T cells in lymph nodes as well as the peripheral circulation which could be used as a surrogate for systemic immunity [35]. Interestingly, the highest concentration of T‐cells at a secondary tumor (simulating a metastatic site) was seen when low dose radiotherapy to the secondary tumor site was combined with hypofractionated radiotherapy to the primary tumor which, in turn, was further amplified by the addition of anti‐PD‐1 immunotherapy. These pathologic findings did translate into significantly better clinical control of the secondary tumor with some showing a complete response [35]. The optimal radiation site for inducing effective immunogenic responses is still largely unknown and, in fact, there may be diminished immunotherapy efficacy dependent upon metastatic site or organ targeted [36].

Other primary inhibitory molecules are implicated in radiation treatment as well. Specifically, radiation can upregulate the expression of PD‐L1 which can be mitigated by combinatorial therapy with an anti‐PD‐L1 agent and has been shown to have superior tumor control compared to either treatment alone [37]. Congruently, dual checkpoint inhibition of CTLA4 and PD‐L1 had optimal tumor response when radiotherapy was administered prior to initiation of immunotherapy in a mouse model [38].

Importantly, these response patterns are maintained when translated into clinical human trials. Secondary analysis of the KEYNOTE‐001 phase I trial investigating single‐agent pembrolizumab (a monoclonal antibody targeting PD‐1) for progressive locally advanced or metastatic NSCLC was performed. When comparing patients who had received radiotherapy prior to immunotherapy to those who received immunotherapy alone, the addition of radiotherapy significantly improved progression‐free survival (PFS) and overall survival (OS) [39]. Interestingly, the median time from any radiotherapy to the first cycle of pembrolizumab was 6.5 months and, when looking at those who received thoracic‐directed radiotherapy, the time from thoracic radiation to the first cycle of ICI was even longer at 11.5 months, thus highlighting that if the result is due to radiation‐induced immunogenicity, it remains unclear the duration of the enhanced response. Baseline patient characteristics on this trial were generally well balanced except for a higher rate of brain metastases and more prior courses of unique systemic therapies in the group having a history of prior radiotherapy while also having a longer time from the initial diagnosis to the initiation of immunotherapy. The longer time interval to beginning immunotherapy may signify the subgroup with a history of radiotherapy may have had a more indolent disease course on this secondary analysis. Taken together, these early preclinical and clinical findings explore the notion that immune checkpoint inhibitor therapy and radiotherapy may cooperate to maximize immunogenic priming and immune targeting of tumor cells leading to additive or possibly synergistic effects.

## 5 Early‐Stage NSCLC

Multiple trials have shown the safety and efficacy of stereotactic body radiotherapy (SBRT; alternatively stereotactic ablative radiotherapy) in patients with early‐stage non‐small‐cell lung cancer [40, 41, 42, 43]. Pooled analysis of two trials comparing SBRT and surgery in operable early‐stage NSCLC demonstrated noninferior survival and potentially lower toxicity with SBRT, though definitive conclusions are difficult to draw considering poor accrual [44, 45]. Given the improvement in local control and overall survival compared to conventionally fractionated radiotherapy, SBRT now represents the standard of care in inoperable patients [46, 47]. While primary tumor control rates exceed 90%, locoregional and distant failure rates each remain above 20% at 5 years [43, 48]. These data underscore the need for improvement in controlling what is likely to be micrometastatic disease that is present during initial definitive management. There is no data showing benefit to the addition of immunotherapy in this setting.

Results of a randomized phase 2 trial comparing the addition of nivolumab to SBRT versus SBRT alone have now been published [49]. Eligible patients included those with treatment‐naive stage IA–IB (tumor size ≤ 4 cm), stage IIA (tumor size ≤ 5 cm), or stage IIB (tumor size > 5 cm and ≤ 7 cm) or isolated recurrences ≤ 7 cm who were unable or unwilling to undergo surgical resection. The majority (80.1%; *n* = 113/141) of patients were newly diagnosed and 87.9% (*n* = 124/141) were ≤ 3 cm (T1). Patients received either 50 Gy in 4 fractions (86.5%) or 70 Gy in 10 fractions (13.5%) and the first dose of nivolumab was to be administered the first day, or within 36 h, of radiation treatment for a total of 4 cycles given every 4 weeks. At 4 years, event‐free survival was improved from 53% to 77% (*p* = 0.0056) favoring the nivolumab arm. This benefit was maintained regardless of PD‐L1 status. The rates of local recurrence (13% vs. 0%), regional recurrence (11% vs. 6%), and distant recurrence (16% vs. 3%) all numerically favored the immunotherapy arm. Grade 3 toxicity was increased from 0% in the SBRT arm to 15% in the nivolumab + SBRT arm, but no grade 4+ toxicities were reported. This represents a meaningful advance in early‐stage NSCLC, though results of ongoing phase 3 studies are needed to validate these findings (see Table 1).

**TABLE 1:** Select ongoing phase 3 trials that are assessing combinatorial radiation and immunotherapy in non‐small‐cell lung cancer.

Registry number (trial name)	Experimental arm	Control arm	Primary endpoint	Expected study completion
SBRT + immunotherapy				
NCT03833154 (PACIFIC‐4) [50]	SBRT + Durvalumab (up to 24 months)	SBRT + placebo	PFS	April 2028
NCT03924869 (KEYNOTE‐867) [51]	SBRT + Pembrolizumab (up to ~12 months)	SBRT + placebo	EFS + OS	July 2026
NCT04214262 [52]	SBRT + Atezolizumab (up to ~6 months)	SBRT	OS	May 2028
Consolidative immunotherapy				
NCT04325763 [53]	sCRT/cCRT + TQB2450 (anti‐PD‐L1) ± anlotinib	sCRT/cCRT + placebo	PFS	March 2025
NCT03706690 (PACIFIC‐5) [54]	sCRT/cCRT + indefinite durvalumab	sCRT/cCRT + placebo	PFS	March 2027
NCT04513925 [55]	cCRT + atezolizumab tiragolumab (up to ~12 months)	cCRT + durvalumab	PFS	December 2027
NCT05221840 (PACIFIC‐9) [56]	cCRT + durvalumab + oleclumab OR monalizumab (up to 12 months)	cCRT + durvalumab	PFS	May 2030
Concurrent immunotherapy				
NCT04092283 [57]	cCRT + concurrent and consolidative durvalumab	cCRT + consolidative durvalumab	OS	December 2028
Concurrent and consolidative immunotherapy				
NCT03519971 (PACIFIC‐2)	cCRT + durvalumab	cCRT + placebo	PFS	October 2024
NCT04380636 (KEYLYNK‐012) [58]	cCRT + pembrolizumab ± olaparib	cCRT + consolidative durvalumab	PFS + OS	July 2026
NCT04026412 (CheckMate73L) [59]	cCRT + nivolumab ± ipilimumab	cCRT + consolidative durvalumab	PFS	December 2026
Radiation boost				
NCT05624996 [60]	cCRT + SBRT boost + consolidative durvalumab	cCRT + consolidative durvalumab	OS + PFS	October 2036

Abbreviations: cCRT, concurrent chemoradiotherapy; EFS, event‐free survival; PFS, progression‐free survival; SBRT, stereotactic body radiotherapy; sCRT, sequential chemoradiotherapy; OS, overall survival.

## 6 Operable Stage III NSCLC

The current standard of care for operable stage III NSCLC includes neoadjuvant chemotherapy with or without immunotherapy followed by surgical resection with the addition of adjuvant radiation and chemotherapy as clinically indicated [46]. The use of immunotherapy in the neoadjuvant setting has significantly benefited patients in multiple randomized trials published in the last year [61, 62, 63, 64]. KEYNOTE‐671 randomized patients with resectable stage II, IIIA, or IIIB to neoadjuvant pembrolizumab or placebo plus cisplatin‐based chemotherapy followed by surgery and adjuvant pembrolizumab or placebo [65]. Overall survival results have now been published, demonstrating an improvement at 36 months from 64% to 71% in those receiving pembrolizumab. This treatment paradigm seems poised to become the standard of care in patients with operable disease. In contrast, neoadjuvant chemoradiotherapy has not shown a consistent survival benefit in resectable stage III NSCLC, though it has demonstrated improvement in pathologic complete response and mediastinal downstaging [66, 67]. With the exceptional advances seen with the addition of neoadjuvant immunotherapy to these patients, it begs the question: could neoadjuvant radiation with immunotherapy or chemoimmunotherapy propel patients' survival to new heights? To our knowledge, there are no reported trials answering this question, but it is the topic of phase 2 evaluation [68]. In the postoperative setting, radiotherapy is not routinely employed after resection in the setting of pathologic N2 disease, though its role has not been clearly defined in the age of neoadjuvant chemoimmunotherapy [69, 70]. As with any neoadjuvant modality, there could be further stratification of patients who may indeed benefit from postoperative treatment.

## 7 Unresectable Stage III Non‐Small‐Cell Lung Cancer

Historically, patients with unresectable stage III NSCLC treated with definitive concurrent chemoradiation have experienced limited progression‐free and overall survival, with median survival typically less than 24 months [71, 72]. The PACIFIC trial delivered, perhaps, the most meaningful improvement in survival for unresectable non‐small‐cell lung cancer of this century [7]. This trial randomized 713 patients undergoing standard chemoradiation to adjuvant durvalumab or placebo. Radiation doses ranged from 54 to 66 Gy, with at least 85% receiving 60–66 Gy. Chemotherapy entailed at least 2 cycles of a platinum‐based regimen with either pemetrexed, paclitaxel, docetaxel, etoposide, vinblastine, or vinorelbine. Median survival in those who received durvalumab was 47.5 months (95% CI, 38.1–52.9) compared to 29.1 months (95% CI, 22.1–35.1) in the placebo arm, and the 5‐year overall survival was improved to 42.9% (95% CI, 38.2–47.4) versus 33.4% (95% CI, 27.3–39.6) [73]. This benefit was present across all PD‐L1 subgroups other than those with < 1% expression. There was no difference in those receiving < 60 Gy compared to 60–66 Gy of total radiation dose [74]. In the 5‐year update, the addition of durvalumab decreased the overall incidence of new lesions from 33.3% to 24.2% [73].

Importantly, patients randomized on the PACIFIC trial had to be free of disease progression prior to enrollment, which makes direct comparison of survival metrics to previous chemoradiation trials difficult given this inherent selection bias. Regardless, adjuvant durvalumab following definitive concurrent chemoradiotherapy now represents the standard of care for patients with unresectable stage III disease and is a significant advancement in unresectable stage III NSCLC. This paradigm will likely shift significantly in the next decade as trials evaluating novel and combination immunotherapies and targeted therapies continue to be published. For instance, phase 2 data from the COAST trial exhibited improvements in 12‐month PFS in those receiving maintenance durvalumab plus oleclumab (anti‐CD73 monoclonal antibody) at 62.6% (95% CI, 48.1–74.2) versus 72.7% (95% CI, 58.8–82.6) with durvalumab plus monalizumab (anti‐NKG2A monoclonal antibody) and 33.9% (95% CI, 21.2 to 47.1) with durvalumab alone following concurrent chemoradiation [75].

In contrast to the benefit seen from consolidative immunotherapy after chemoradiation in this population, phase III data from the PACIFIC‐2 trial has been presented in abstract form demonstrating no benefit with the addition of concurrent immunotherapy [76]. Patients with stage III, unresectable non‐small‐cell lung cancer were randomized to concurrent chemoradiation alone or with concurrent and consolidative durvalumab with a primary endpoint of progression‐free survival. There were 327 patients randomized, 219 to the durvalumab arm, and 108 to the placebo arm. Ultimately, there was no difference in PFS, which was 13.8 versus 9.4 months (HR, 0.85; 95% CI: 0.65–1.12; *p* = 0.247). Median overall survival was similarly not improved, with a median OS of 36.4 versus 29.5 months (HR, 1.03; 95% CI: 0.78–1.39; *p* = 0.823). Of note, these results are similar to studies in patients with small‐cell carcinoma of the lung [77, 78]. These data suggest the timing of administration of immunotherapy in relation to radiotherapy delivery plays a crucial role in response to treatment, with concurrent administration exhibiting less favorable results.

Additional phase 3 data include the GEMSTONE‐301 randomized multicenter trial conducted in China with preliminary data reported [79]. This study randomized 381 patients with locally advanced, unresectable NSCLC after receiving either sequential *or* concurrent platinum‐based chemoradiation to placebo or maintenance sugemalimab, a novel PD‐L1 antibody with enhanced immunologic tumor presentation activity. More than 50% of patients had stage IIIB disease and two‐thirds of both arms received concurrent chemoradiation with most patients receiving ≥ 60 Gy. Median PFS was 9.0 versus 5.8 months (*p* = 0.0026) favoring the sugemalimab arm. Long‐term results are awaited. There are additional phase 2 trials supporting the safety and efficacy of concurrent or consolidative immunotherapy after definitive chemoradiation for unresectable stage III NSCLC [80, 81, 82].

In more frail patients, there can be concern regarding the tolerance of concurrent chemoradiation leading to the clinical decision to pursue sequential therapy. PACIFIC‐6 is a phase 2, nonrandomized trial evaluating safety of durvalumab following sequential chemotherapy and radiotherapy [83]. This trial enrolled 117 patients with unresectable stage III NSCLC who had received platinum‐based chemotherapy followed by radiotherapy (60 Gy ± 10%) with no progression at restaging to then receive up to 24 months of maintenance durvalumab. Rates of grades 3 and 4 toxicity were similar to those observed in the PACIFIC trial [7]. Survival data were immature at publication, but median PFS was 10.9 months (95% CI: 7.3–15.6) with 12‐month PFS and OS of 49.6% and 84.1%, respectively.

As evidenced by ongoing clinical trials, the timing of immunotherapy with regards to chemoradiation remains an unanswered question. As previously discussed, given the biologic interplay of radiation and immunotherapy, a reasonable estimation would be the two delivered sequentially—with immunotherapy following radiotherapy—may lead to optimal efficacy. Indeed, a meta‐analysis investigating combination therapy of radiation with PD‐1/PD‐L1 inhibitors compared with noncombination therapy demonstrated improvement in 2‐year PFS (OR 2.47, 95% CI: 1.13–5.37, *p* = 0.023), 2‐year OS (OR 1.77, 95% CI: 1.35–2.33, *p* = 0.000) [84]. In this same study, subanalysis demonstrated receipt of radiotherapy followed by immunotherapy outperformed groups in which immunotherapy was given prior to radiation. There are multiple ongoing phase 3 trials assessing the utility of induction, concurrent, or consolidative immunotherapy, as listed in Table 1.

## 8 Metastatic Non‐Small‐Cell Lung Cancer

Chemotherapy comprises the backbone of metastatic non‐small‐cell lung cancer treatment in the absence of targetable genomic alterations [46]. The addition of immunotherapy to chemotherapy has improved survival in those with metastatic NSCLC, though median survival remains modest, with median PFS of around 7–9 months and median overall survival approaching 20 months [5, 24, 85, 86]. There have been encouraging findings regarding the use of stereotactic body radiotherapy (SBRT) in combination with immunotherapy in patients with metastatic disease.

The PEMBRO‐RT trial was a phase 2 study randomizing 76 patients with metastatic NSCLC to pembrolizumab with or without radiotherapy (24 Gy in 3 fractions) to a single tumor site with a primary endpoint of overall response rate (ORR) [22]. Although there was an improvement in ORR, 18% in the control arm versus 36% in the experimental arm (*p* = 0.07, alpha = 0.10), the cutoff for trial positivity was not met; however, in the PD‐L1‐negative subgroup, overall survival was improved (hazard ratio [HR], 0.48, *p* = 0.046) along with PFS (HR, 0.49, *p* = 0.03). Importantly, there was no increase in toxicity. Similar results were seen in a phase I/II trial from MD Anderson, in which 80 patients with metastatic non‐small‐cell lung cancer received pembrolizumab with or without radiotherapy [87]. Radiotherapy could either be SBRT (50 Gy in 4 fractions) or hypofractionated (45 Gy in 15 fractions). In those in the radiotherapy arm, 90% (36/40) received radiation to one site, whereas the remaining 10% received radiation to two sites. The primary endpoint, out‐of‐field response rate (ORR), was not significantly different, 22% versus 25% (*p* = 0.99). On exploratory analysis, those receiving SBRT demonstrated an improvement in median PFS, 20.8 versus 6.8 months (*p* = 0.03). Furthermore, those with low PD‐L1 expression demonstrated improvement in median PFS times, increasing from 4.6 to 20.8 months (*p* = 0.001), whereas the benefit was lost when including all patients. These findings could illustrate ablative radiotherapy more effectively modulates the immune microenvironment in tumors that are PD‐L1 low; however, definitive conclusions are unable to be drawn given the low numbers of patients in these exploratory analyses.

A pooled analysis of the two aforementioned trials demonstrated an improved out‐of‐field (abscopal) response rate (ARR) from 19.7% to 41.7% (odds ratio [OR] 2.96, 95% CI 1.42–6.20; *p* = 0.0039) in those receiving pembrolizumab with radiotherapy [88]. There were benefits in median PFS, 4.4 versus 9.0 months (HR, 0.67, 95% CI 0.45–0.99; *p* = 0.045) and overall survival, 8.7 months versus 19.2 months (HR, 0.67, 0.54–0.84; *p* = 0.0004) in those receiving radiotherapy as well. Once again, exploratory analysis showed a PFS benefit in those with low‐PD‐L1 status (HR 0.40, 95% CI 0.16–0.96; *p* = 0.012). While not statistically significant, the ARR was highest in those receiving either 50 Gy in 4 fractions (56.2%; 9/16 patients) or 24 Gy in 3 fractions (46.2%; 17/36 patients) compared to those who received 45 Gy in 15 fractions (20%; 4/20 patients). Intriguingly, they found significant lymphocyte depletion only in those patients receiving 45 Gy in 15 fractions compared to the SBRT regimens, potentially highlighting an anti‐immunogenic effect. These findings are thought‐provoking and should serve as the basis for future trials evaluating the effect of radiation dose in these patients. The ongoing NIRVANA‐Lung phase 3 trial includes patients with stage IIIB‐IV NSCLC patients receiving chemotherapy and pembrolizumab with or without radiotherapy (at least 18 Gy in 3 fractions) with the primary endpoint of overall survival [89].

Additional applications for combining immunotherapy and radiotherapy would certainly be in those with oligometastatic NSCLC. SBRT to metastatic sites has been increasingly utilized in this setting either alone or with systemic therapy. Trials have demonstrated a progression‐free and overall survival benefit of local consolidative therapy to metastatic sites compared to maintenance systemic therapy alone in those with ≤ 3 metastatic lesions without progression after first‐line chemotherapy, though these trials were performed in the preimmunotherapy era [90, 91]. Similar improvements have been illustrated in those with ≤ 5 metastatic lesions and in the oligoprogressive setting [92, 93]. Further research is needed to assess the potential benefit of local consolidative therapy in the setting of immunotherapy.

## 9 Conclusions

Combinatorial radiation and immunotherapy in patients with non‐small‐cell lung cancer is a burgeoning field. Current evidence discussed above has shed light on the benefits seen in early, advanced, and metastatic non‐small‐cell lung cancer (see Table 2 for a summary of select reported phase 2/3 trials previously discussed). The recently published data showing improvements in event‐free survival for those with early‐stage NSCLC receiving concurrent and consolidative nivolumab after SBRT are particularly noteworthy as this only serves to fuel the comparison to surgery in this setting. Ongoing trials are currently evaluating different immunotherapy regimens and length of treatment (Table 1) [49].

**TABLE 2:** Select reported phase 2/3 trials combining immunotherapy and radiation therapy in non‐small‐cell lung cancer.

Registry number (trial name)	Phase	Stage	Experimental arm	Control arm	Outcome
SBRT + immunotherapy					
NCT03110978 [49]	Phase 2	IA–IIB or recurrent ≤ 7 cm	SBRT + nivolumab	SBRT	4‐year EFS: 77% versus 53% hazard ratio (HR, 0.38; 95% CI: 0.19–0.75)
Concurrent and consolidative immunotherapy					
NCT03519971 (PACIFIC‐2) [76]	Phase 3	III	cCRT + durvalumab	cCRT + placebo	Median PFS: 13.8 versus 9.4 months (HR, 0.85; 95% CI: 0.65–1.12)
Consolidative immunotherapy					
NCT02125461 (PACIFIC) [73]	Phase 3	III	cCRT + durvalumab (up to 12 months)	cCRT + placebo	Median OS: 47.5 versus 29.1 months (HR, 0.72; 95% CI: 0.59–0.89)
NCT03728556 (GEMSTONE‐301) [79]	Phase 3	III	cCRT/sCRT + sugemalimab (up to 24 months)	cCRT/sCRT + placebo	Median PFS: 9.0 versus 5.8 months (HR, 0.64; 95% CI: 0.48–0.85)
Immunotherapy ± SBRT					
NCT02492568 (PEMBRO‐RT) [22]	Phase 2	IV	Pembrolizumab + SBRT	Pembrolizumab	12 week ORR: 36% versus 18% (*p* = 0.07)
NCT02444741 [87]	Phase 1/2	IV	Pembrolizumab + RT	Pembrolizumab	ORR: 22% versus 25% (*p* = 0.99)
Pooled analysis of NCT02492568 and NCT02444741 [88]	N/A	IV	Pembrolizumab + RT	Pembrolizumab	ARR: 41.7% versus 19.7% (OR, 2.96, 95% CI: 1.42–6.20)

Abbreviations: ARR, abscopal response rate; cCRT, concurrent chemoradiotherapy; EFS, event‐free survival; HR, hazard ratio; OR, odds ratio; ORR, objective/overall response rate; OS, overall survival; PFS, progression‐free survival; RT, radiation therapy; SBRT, stereotactic body radiotherapy; sCRT, sequential chemoradiotherapy.

Perioperative radiotherapy is not routinely employed but will need to be reevaluated in the setting of neoadjuvant chemoimmunotherapy. Preoperative chemoradiation has demonstrated improvements in pathologic complete response, and research evaluating trimodality neoadjuvant treatment (radiotherapy, chemotherapy, and immunotherapy) followed by resection is ongoing [68]. The benefit of combinatorial therapy has been clearly defined in those with unresectable stage III disease since the results of the PACIFIC trial [7, 73, 74]. We would be remiss to fail to acknowledge the receipt of chemotherapy confounds the true relationship between radiotherapy and immunotherapy in these trials, but ongoing trials will further delineate the optimal timing of immunotherapy in this setting, whether it be induction, concurrent, or consolidative. This will help to elucidate the relationship between radiation and immunotherapy in these patients.

There is early evidence showing the safety and efficacy of combinatorial radiation and immunotherapy for metastatic NSCLC and the optimal use of radiotherapy with immunotherapy in the oligometastatic setting remains a stirring question [88]. There are still unanswered questions within this population. For instance, what is the optimal dose of radiation to augment the immunogenic response and efficacy of immunotherapy? What is the optimal site and a number of tumors irradiated in those with metastatic disease—could low‐dose radiation to *all* metastatic sites improve variation of antigenic presentation and thus enhance the local and distant response to immunotherapy? These and more will serve as a basis for future research in this area. However, regardless of the questions that remain, combinatorial radiation and immunotherapy has proven beneficial in patients with NSCLC and seems poised to deliver numerous breakthroughs in the near future.

## Author Contributions

**Justin L. Burr:** conceptualization (equal), formal analysis (equal), investigation (equal), methodology (equal), project administration (equal), writing – original draft (equal), writing – review and editing (equal). **Kurtis C. Johnson:** conceptualization (equal), formal analysis (equal), investigation (equal), methodology (equal), project administration (equal), writing – original draft (equal), writing – review and editing (equal). **Joseph J. Carmicheal:** conceptualization (equal), investigation (equal), methodology (equal), project administration (equal), writing – original draft (equal), writing – review and editing (equal). **Chi Lin:** conceptualization (equal), investigation (equal), project administration (equal), supervision (equal), writing – review and editing (equal). **Apar Kishor Ganti:** conceptualization (equal), methodology (equal), project administration (equal), supervision (equal), validation (equal), writing – review and editing (equal).

## Conflicts of Interest

The authors declare no conflicts of interest.

## Data Availability

Any research data are stored in an institutional repository and will be shared upon request to the corresponding author.
